# Awareness of Iranian’s General Dentists Regarding the Latest Prophylaxis Guideline for Prevention of Infective Endocarditis

**Published:** 2013-03

**Authors:** F Ghaderi, M Oshagh, R Dehghani, R Hasanshahi

**Affiliations:** a Dept. of Pedodontics, School of Dentistry, Shiraz University of Medical Sciences, Shiraz, Iran; b Dept. of Orthodontics, Member of Orthodontic Research Center, School of Dentistry, Shiraz University of Medical Sciences, Shiraz, Iran; c Dentist, Member of Student Research Committee, Shiraz University of Medical Sciences, Shiraz, Iran

**Keywords:** Antibiotic Prophylaxis, Endocarditis, Knowledge, Dentists

## Abstract

**Statement of Problem** **:** Dental procedures leading to oral tissue injuries may provoke bacterial release to the blood stream causing infective endocarditis (IE) in vulnerable patients. The guideline which was proposed by AHA has been updated 9 times having the last update published in 2007. This study was endeavored to uncover the level of knowledge of general dental practitioners in Shiraz, concerning the 2007 AHA guidelines for endocarditis prophylaxis in patients with cardiac problems receiving dental treatments.

**Materials and Method:** This cross- sectional and descriptive analytical study included 150 dentists as participants. All practitioners were given a self –report questionnaire which consisted of three sections. Questions were designed to assess their knowledge of antibiotic prophylaxis in patients with cardiac disease.

**Results:** Almost all participants (93%) were aware of antibiotic prophylaxis to be essential for tooth extraction. Most participants did not believe in prophylaxis for noninvasive procedures (such as shedding of primary teeth, impression, intraoral radiography). From all of the respondents, 75% considered Amoxicillin to be the antibiotic of choice and 57% were acquainted with the correct dose of Amoxicillin for high risk patients.

**Conclusion:** The study identified a potential for under/over prescription of antibiotic prophylaxis under the current guideline. Burden of IE necessitates more accurate knowledge of antibiotic prophylaxis in the undergraduate curriculum and continuing education programs of dentistry.

## Introduction

Infective endocarditis (IE) is defined as an exudative and proliferative inflammatory alteration of the endocardium resulting from bacteremia [1]. This is a rare disease resulted in mortality and morbidity in 10-80% of patients [1-3]. Repeated attacks of endocarditis reduce the 5-year survival of patients to 60% [4]. IE is difficult to treat and has a poor prognosis. Thus, an understanding of the various prophylactic measures is of utmost importance in dealing with this disease [4]. 

Subacute endocarditis, the most common subtype, occurs following dental procedures in susceptible patients [4]. The oral cavity harbors various micro-organisms that can enter the blood circulation through tissue injury [5-8]. With increased survival rate of patients with heart diseases [9-10], dental practitioners are more likely to be involved in dental treatment of patients with cardiac diseases [11-12]. Dental manipulations including oral surgery, periodontal procedures and root canal therapy can lead to infection of sterile vegetations on cardiac valves in susceptible patients to endocarditis. The main consideration in their dental management of patients has been the prerequisite of appropriate antibiotic prophylaxis (AP) when clinically indicated [5-7, 13-14]. 

While some dental procedures and some specific cardiac problems are well recognized to be a clear indication for AP in prevention of (IE); there is some controversy over other dental treatments as to whether or not they need prophylaxis [13]. For this reason, relevant guidelines have been published by a number of bodies and have been updated periodically in many years [11-12, 15]. Expert groups, like the American Heart Association (AHA) issue guidelines periodically to specify antibiotic regimens for prophylaxis and its indications [11, 16]. The AHA and European guidelines highlighted the importance of controlling the microbial in professional oral health cares and daily oral home care which reduces possible bacteraemias [17-18]. A systematic review, conducted by the Cochrane collaboration in 2005 stated that there was a lack of evidence to support published guidelines [19]. 

Since 1955, the guideline of AHA has been updated 9 times, with the last update published in 2007. The American Heart Association (AHA) states that the guideline was developed through an evidence-based approach [20]. The 2007 guidelines state that antibiotic administration is reasonable before all dental procedures which involve manipulation of the gingival tissue or the peri-apical area of teeth or the oral mucosa for patients at risk. The updated AHA recommendations clearly state that most cases of IE are not concerned to invasive dental procedures and suggested antibiotics have not been changed since 1997 [20]. Either inadequate knowledge or poor agreement with previous guidelines has been reported in patients and health care providers [21-24]. Many dentists are confused regarding the indication or the type of antibiotic prophylaxis. They often rely on recommendations from cautious practitioners who rely on their own individual experiences and suggest prophylaxis in any unpromising situations [1]. 

The aim of this study was to determine the level of awareness of GDPs in Shiraz, south of Iran, regarding implementations of the 2007 AHA guidelines for endocarditis prophylaxis in cardiac patients receiving dental treatments through questionnaires. 

## Materials and Method

This was a cross-sectional descriptive analytical study included 150 GDPs as participants in Shiraz- Iran. There are approximately 311 general dental practitioners in Shiraz (2007 and 2008), of whom 150 were randomly selected for this study as recommended by the statistical consultant. From all the questionnaires, 80 questionnaires were answered and the results were collected. The Ethics committee at Shiraz University of Medical Sciences approved the study design.

In 2007 and 2008, 150 dentists were requested to participate in the study and to complete a three-page questionnaire. The self-reporting questionnaire consisted of three parts to investigate the proper indications in which general dental practitioners would prescribe prophylactic antibiotics and the regime used: 1) Knowledge of which cardiac condition requires AP. (Q1) 2) Knowledge of which dental procedure requires AP. (Q2) 3) The AP regime they used. (Q3) 

The questionnaire included closed questions with only one correct answer for each question. These questions were based on the latest endocarditis prophylaxis regimens recommended by the AHA in 2007. 

Questionnaires were designed, without asking the name of participants and were given to the dentists to complete. The questionnaires were given to the dentists with enough explanation. The level of relative knowledge of the dentists was scored base on the number of correct answers for each section.

 The questionnaire also collected demographic information including participant’s gender and duration of their practical experience. 

For part 1 and 2, the percent of questions in each section which was answered correctly by each respondent, was calculated to derive a summary score.

The responses were entered into the statistical package for social science (SPSS Inc.) database and the overall response rate and percentage responses for each question were calculated. 

For statistical analysis correlation Pearson and Student t test were used. The differences between males and females were evaluated using Student’s t-test. The association between the work experience and the questionnaire scores of general dental practitioners with their knowledge was assessed using Pearson correlation.

## Results

From 150 questionnaires, 80 questionnaires (53.4%) were returned. Table1 presents the demographic characteristics of the participants. The level of acquaintance on 2007 AHA guidelines was 56 % (7.41%- 85.19%). 

**Table 1:** Demographic details of the study participants

**Characteristic**		
No. of participants		80
Gender, n	Male Female	54(67.5%) 26(32.5%)

The overall level of knowledge among male and female dentists was 60.4% and 46.8% respectively which shows that male dentists had significantly higher level of knowledge than female dentists (*p*= 0.01).

The responses to the questionnaire showed 85 percent of respondents were aware that valve replacements required antibiotic prophylaxis.

Concerning imperative medical conditions, only 5% of participants were aware of requisite antibiotic prophylaxis for MVP with valvular regurgitation while 38% recognized its crucial need in bypass graft surgeries (Table 2).

**Table 2:** Proportion of correct responses for each heart condition

**Heart conditions**	**Correct answers (%)**
MVP with valvular regurgitation	5.0
Patent ductus arteriosus	13.8
Previous Rheumatic fever	22.5
Atrial septal defect	28.8
Coronary artery bypass graft surgery	38
Pacemaker	50.0
MVP without valvular regurgitation	61.3
Valve replacements	85

93 percent of participants indicated prophylaxis need in tooth extractions, while most of participants did not point to prophylaxis necessity in noninvasive dental procedures such as shedding of primary teeth, impression and intraoral radiography.

On the other hand, almost half of the respondents answered correctly concerning other dental treatments such as restoration of caries lesion, insertion of rubber dam, placement of banded orthodontic appliance and local anesthetic infiltration (Table 3). 

From all of the respondents, 75% believed to prescribe Amoxicillin as the antibiotic of choice and 57% have been aware of the correct dose of Amoxicillin for high risk patients.

Mean value of work experience was 8.7 years 

(maximum 30 years and minimum 1 year) and the correlation between work experience and the level of knowledge was reverse and significant (Pearson correlation= -0.291, *p*= 0.009).

**Table 3:** Proportion of correct responses for each dental treatment

**Dental procedures**	**Correct answers (%)**
Tooth extraction	93
Periodontal surgery	73
Shedding of primary teeth	83
Oral impression	82
Restoration	58
Rubber dam insertion	56
Orthodontic band placement	77.5
Orthodontic adjustment	36.3
Fluoride therapy	85
Local anesthetic infiltration	58
Intraoral radiographs	83
Periodontal procedures	92.5

## Discussion

In this study, the overall level of relative awareness of dentists regarding implementations of the 2007 AHA guidelines for endocarditis prophylaxis in cardiac patients receiving dental treatments was approximately 56% which is comparable to results of Eskandari et al. (59%) [5]. Boyle et al. demonstrated that 56% of GDPs in Ireland were aware of that latest prophylaxis regimen which is similar to the results of this study [25]. In Hashemipour et al.’s study, the knowledge of dentists about prescribing antibiotics for prevention of bacterial endocarditis was relatively low (less than 50%). Our result is lower than the results obtained by Chitsazi and Pourabbas (76.2%) [26]. The level of knowledge of the 2007 AHA guidelines in this study was less than results of Zadik et al. regarding the 2007 guidelines (81.3%) [27] and Epstein et al. regarding the 1997 guidelines (>80%) [28]. This was similar to the findings related to the earlier versions of the AHA guidelines (29%-81%) [21-24]. Epstein et al. reported that 17.5 percent of respondents were not using the current guidelines for prophylaxis against infective endocarditis. Most of the dentists in their study were using the early 1997 dose recommendations [28]. In Palmer et al.’s study a high proportion of the GDPs followed current guidelines on AP for patients with cardiac problems [29] Van der Meer and colleagues reported that there was poor awareness of recommendations for prophylaxis among health care provider [30]. 

In this study, the least awareness (38% correct answers) was evident in necessity the AP for patients undergone bypass graft. Also the most prominent inadequate awareness was detected regarding the MVP with valvular regurgitation. This is in agreement with Zadik et al.’s results [27]. These patients were in the IE moderate risk group in the 1997 regimen, but not in the 2007 update. In current guideline, AHA recommends antibiotic prophylaxis for MVP patients only when regurgitation exists [31]. 

In this study the responses to the questionnaire showed that most dentists (85%) were aware that valve replacements required antibiotic prophylaxis. This is in agreement with the results of Thompson et al.’s study [13]. In Hashemipour et al. study 94.6% of all answers on prescribing of antibiotic in patients with prosthetic cardiac valves were correct [32]. It must be stated that the AP guidelines considered in this paper, reflect the newest version of guideline at the time of the distribution of the questionnaire in 2007. 

In this study, the percentage of participants who prescribe antibiotics before tooth preparation with oral impressions and restoration of a caries lesion in high- risk patients was 18% and 42% respectively. These percentages were 62% and 23% in Zadik et al. study [27]. The possibility and risk of gingival involvement during different phases of endodontic, restorative, and prosthodontic procedures may lead to ambiguity among practitioners [27]. In addition, procedures that require administration of antibiotics should be listed in detail in all educational programs.

In high risk patients, the percentage of participants who prescribe antibiotics before tooth extractions was 93%. This is a rationale procedure to prescribe antibiotic for extractions, since tooth extraction is thought to a dental procedure causing bacteremia most likely, with an incidence ranging from 10 to 100 percent [33]. In Palmer et al.’s study a large proportion of the respondents prescribed prophylactic antibiotics for surgical extractions (39%) [29]. It must be remembered that risk of IE is related both to the type of the dental treatment and the nature of the cardiac defect [13]. 

In this study, 77.5 % of dentists answered correctly to the question which concerned the requisite AP before orthodontic band placement. Current recommendations advise that orthodontic bands entail AP whilst bracket placement does not [13, 34]. 

In the present study, male dentists had significantly higher level of knowledge than female dentists (*p*= 0.01). This is not in agreement with the results of Eskandari et al. [5] and Chitsazi et al. [26] which found no difference between awareness of two genders. Fakhrayi et al. found that female dental students had a higher knowledge of cardiac diseases, dental procedures and prophylaxis regimen than males [5, 26]. This might be attributed to the more conservative feeling of the females.

In this study, the association between the practice period and the level of knowledge was significant. This is in agreement with findings of Eskandari et al., Hashemipour et al., Lauber et al. and other studies which showed that the level of knowledge in current graduates was more than previous graduates [5, 25, 28, 32, 35-36]. Epstien et al. observed that current graduates prescribed prophylactic antibiotics at a lower rate than former graduates. They concluded that undergraduate education or continuing education programs might be successful in informing dentists about current antibiotic practices [28]. 

AP is provided to prevent the development of bacterial endocarditis as a consequence of odontogenic bacteraemia [37]. Two mechanisms are thought to be responsible. First a reduction in the numbers of organisms in the blood and second a reduction in the adhesion of organisms to the non-bacterial thrombotic vegetation [38]. For 50 years, the AHA has recommended penicillin as the preferred choice for dental prophylaxis for IE. Now it is believed that a single dose of Amoxicillin or Ampicillin is safe and is the prophylactic choice for patients [36]. Amoxicillin is well- absorbed in the gastrointestinal tract and provides high and constant serum concentrations [40]. Recent studies suggest that Amoxicillin therapy has a significant impact on reducing the incidence, nature and duration of bacteraemia from dental procedure does not eliminate it [41]. The most notable change in protocol includes reduction of the oral dose of Amoxicillin from 3 grams to 2grams, with no follow- upping dose [11, 42]. Standard prophylactic regimen for certain dental procedures is 2.0 grams Amoxicillin orally 30-60 minutes before procedure [11, 40]. Dajani and colleagues have reported that 2 grams of Amoxicillin provides several hours of antibiotic cover age [43]. 

In our study more than half (57%) of respondents believed the appropriate dose to be 2 grams Amoxicillin before treatment. From all of the respondents, 75% prescribed Amoxicillin as an antibiotic of choice and 57% prescribed correct dose of Amoxicillin for high risk patients. In Hashemipour et al.’s study more than half of the dentists (62.8%) had chosen Amoxicillin as a prophylactic antibiotic [32]. In Palmer et al.’s study a single three-gram dose of Amoxicillin was the choice of prophylactic antibiotic coverage, provided by 90.6% of the respondents; a two-gram dose regimen of Amoxicillin was used by 9.2% of respondents [29]. 

The main shortcoming of the present study might be the small number of participants (80 persons). These Individuals may not represent all dentists, but they can be referred as a substantial source for dental treatments of patients with cardiac problems.

Early researches have revealed that a concise recognition of the guidelines for endocarditis prophylaxis in cardiac patients, receiving dental treatments, may have been overlooked by dental professions, during their education [44]. This negligence in dental education seems to have aroused from many reasons such as insufficient information about patient’s cardiac disease or even concerning these protocol‘s updates to be unneeded [20, 45]. Revisions in the dental curriculum to cover AP for bacterial endocarditis as well as additional educational programs in the form of posters, brochures, and continuing education programs for graduates should be introduced. This will improve acquaintance of GDPs about dental considerations in medically- compromised patients [5]. We suggest further clarification and education of the current antibiotic guidelines. 

However, further large scale surveys among dentists, are warranted. Since any recommendations for IE prophylaxis must be evidence- based; a placebo- controlled, multicenter, randomized, double- blinded study with a large number of patients and professionals is needed to evaluate the efficacy of prophylaxis in susceptible patients [3]. 

## Conclusion

The results of the current study revealed an average level of awareness GDPs in Shiraz regarding endocarditis prophylaxis. The study identified potential for under- and over- prescription of AP under the current guideline. Burden of infective endocarditis necessitates more accurate knowledge of antibiotic prophylaxis in the undergraduate curriculum and continuing educational programs for graduate dentists.
